# Sertoli-Leydig Cell Tumor with Concurrent Rhabdomyosarcoma: Three Case Reports and a Review of the Literature

**DOI:** 10.1155/2017/4587296

**Published:** 2017-07-02

**Authors:** Trisha Plastini, Arthur Staddon

**Affiliations:** Pennsylvania Hospital, Penn Medicine, Philadelphia, PA, USA

## Abstract

Sertoli-Leydig Cell Tumors (SLCTs) make up <1% of all ovarian tumors and are benign or malignant, androgen-secreting tumors. Rhabdomyosarcoma (RMS) is a heterogeneous group of malignant tumors that resemble developing skeletal muscle. There have been case reports of patients with concurrent SLCT and RMS with limited treatment options. We aim to demonstrate treatment strategies used in our patients, which seemed to have prolonged survival when compared to prior case reports of patients not cured by surgical resection. Herein we describe 22 cases of SLCT with RMS elements as discussed in prior case reports and three cases from the authors' institution. Of the 19 cases from prior case reports, five were lost to follow-up and two had NED after surgical intervention. Eleven patients had recurrence and were deceased within one year. Of those patients not surgically cured, only three patients were documented as living beyond two years, all of whom received chemotherapy. The three patients presented from our institution had clinical evidence of response to chemotherapy that is traditionally used for RMS. In conclusion, chemotherapy with doxorubicin and ifosfamide has activity in patients with SLCT and RMS as does salvage chemotherapy with vincristine, irinotecan, and temozolomide.

Sertoli-Leydig Cell Tumors (SLCTs), also known as arrhenoblastomas, make up <0.5–1% of all ovarian tumors and are benign or malignant, androgen-secreting tumors originating from the ovarian stromal sex cords [1]. In up to half of cases, SLCT can be associated with signs of virilization, such as hirsutism and amenorrhea, and SLCTs occur more often in women of reproductive age [1, 2]. There are five histological subtypes of SLCT as follows: well differentiated, intermediate differentiation, poorly differentiated, retiform, and heterologous or mixed [3–5]. Twenty percent of SLCTs are variants with heterologous elements, which are mostly benign gastrointestinal epithelium, but 5% of SLCTs contain heterologous mesenchymal elements [4]. Ultrasound is the primary imaging modality to identify adnexal masses. Serum levels of AFP and *β*-hCG may be elevated in patients with SLTC [2]. SLTC immunocytochemical characterization is positive for testosterone and estradiol of the Sertoli and Leydig cells [1]. A mutation of the DICER1 gene has been associated with SLCTs in up to 63% of patients, and patients with this mutation should be screened for thyroid disease [6, 7]. The immunohistochemical testing of antibodies against alpha-inhibin and myogenin is needed to diagnose rhabdomyosarcoma (RMS) and accurately grade the SLCT [8]. The TMN and FIGO stages are listed in Table 1. The treatment and prognosis of SLCT depend on the patient's age, tumor stage, and degree of tumor differentiation [1, 2]. Various types of surgery with or without adjuvant chemotherapy are the primary treatment modalities for treatment of SLCT [2]. Malignant SLCT ten-year survival rates are approximately 87% for intermediate differentiation and 41% for poor differentiation [9].

Rhabdomyosarcoma (RMS) is a heterogeneous group of malignant tumors that resemble developing skeletal muscle [10–12]. These tumors are 1.4 times more common in males without differences among races or ethnic groups [13]. RMS is the most common soft tissue sarcoma of childhood, and common sites of primary disease include the head and neck area, genitourinary tract, and extremities [10, 11]. Most cases of RMS appear to be sporadic, but the disease has been associated with familial syndromes such as Li-Fraumeni syndrome and neurofibromatosis [10]. There are two histologic subtypes of RMS, embryonal and alveolar; Alveolar RMS is more aggressive with small, round cells while embryonal RMS has a more favorable prognosis with spindle-shaped cells with a stromal-rich appearance [10, 11, 14]. Embryonal variants include leiomyomatous RMS that is predominantly of paratesticular origin and the botryoid variant with subepithelial aggregates of tumor cells known as the cambium layer [14]. Sarcoma botryoides can arise from the mucosal surfaces of the cervix, bladder, or vagina [15]. Radiologic evaluation of RMS should include CT scan or MRI of the primary and surrounding structures. Treatment approaches to RMS incorporate chemotherapy, radiation therapy, and surgery based on risk stratification. Complete surgical resection is considered if it will not be cosmetically damaging. The five-year overall survival rate for adults with RMS is approximately 27% [16].

The association between cervical sarcoma botryoides and ovarian SCLT has been described in at least eight patients from prior case reports [15, 17–22]. Adjuvant ifosfamide and epiadriamycin were used in one of these patients who had recurrence of uterine disease after 6 years, and conservative polypectomy was encouraged by some authors [20]. A genetic linkage to abnormalities on chromosome 12 was suggested by one paper [19]. One patient with concurrently diagnosed pleuropulmonary blastoma and embryonal rhabdomyosarcoma of the cervix had a DICER1 germline mutation [21]. This patient was treated with vincristine, actinomycin, and cyclophosphamide with recurrence of SLCT 7 years later [21].

Herein, we report three cases of patients with ovarian SLCT with RMS components. To our knowledge, there have been cases reported in literature with limited treatment options. We aim to demonstrate treatment strategies used in our patients, which seemed to have prolonged their lives when compared to prior case reports of patients not cured by surgical resection, in order to provide treatment options for clinicians treating patients with these concurrent rare malignancies.

Patient 1 was a 32-year-old Caucasian female noted to have a left sided ovarian mass on CT scan after presenting for left lower quadrant abdominal pain. She was status post resection and pathology was consistent with stage Ia poorly differentiated SLCT. Five months later, recurrent pains lead to a trans-vaginal US that showed a right pelvic mass. She underwent radical hysterectomy and debulking procedure that showed pathology consistent with recurrent poorly differentiated SLCT with heterologous RMS. A follow-up PET scan showed uptake bilaterally in the pelvis. A month later, she was started on doxorubicin, ifosfamide, and vincristine. She was hospitalized for a small bowel obstruction with stable metastatic abdominal nodules on CT. She received cycle 2 doxorubicin, ifosfamide, and vincristine while hospitalized and had a palliative PEG placed. Her chemotherapy was changed to ifosfamide and etoposide because of mucositis and palmar-plantar erythrodysesthesia. After a total of 6 cycles of chemotherapy, she was found to be in complete clinical remission by CT. Approximately 10 months later, CT showed a liver lesion and pelvic mass consistent with recurrent poorly differentiated SLCT on repeat biopsy with no RMS elements seen. She was given 3 cycles of bleomycin, etoposide, and cisplatin (BEP). Bleomycin was held for cycles 4 through 8 due to side effects. A follow-up PET showed stable disease. Three months later, she was found to have a left lower quadrant mass consistent with recurrent SLCT at which point radiation therapy was given with good response with only a small amount of residual disease. Eight months later, a biopsy of a liver lesion was consistent with recurrent SLCT with no definitive RMS identified. She underwent further radiation with stable disease for approximately five months before repeat imaging showed marked disease progression with symptoms that lead to hospice care. The time from diagnosis until death was approximately four years.

Patient 2 was a 30-year-old, black female with a left sided ovarian mass found incidentally during a cesarean section that was initially called a granulosa tumor on pathology but later deemed SLCT with focal poor differentiation when reviewed. At the time of initial diagnosis she was thought to have stage IA disease that was cured surgically. She was lost to follow-up for 13 months at which time a follow-up CT showed a right sided ovarian mass that increased on serial imaging. Surgery for recurrence revealed a left adnexal mass, left pelvic sidewall, cul-de-sac, and anterior abdominal wall nodules with pathology consistent with high-grade sarcoma ultimately deemed embryonal RMS consistent with recurrence of the poorly differentiated SLCT. Two months later, she was started on doxorubicin, ifosfamide, and vincristine for a total of 6 cycles with follow-up CT showing NED. A repeat CT performed 7 months after chemotherapy completion showed an increasing pelvic mass that was resected. Pathology was consistent with recurrent SLCT with RMS, and a liver mass was discovered during surgery that was not seen on CT scan. She was started on salvage chemotherapy for RMS with vincristine, irinotecan, and temozolomide for a total of 6 cycles. Follow-up CT after cycle 4 showed NED. The patient developed massive progression 5 months after her last cycle of chemotherapy with small bowel obstruction requiring debulking surgery with colostomy placement and ultimately hospice care. The time span from diagnosis until death was approximately 3.5 years.

Patient 3 was a 23-year-old female who presented with nausea and vomiting with an US showing a right ovarian mass. She underwent exploratory laparotomy with debulking and removal of a large abdominal pelvic mass with a right salpingo-oophorectomy (RSO) and appendectomy. Pathology was consistent with stage IA poorly differentiated SLCT with some elements of RMS, and she was found to have a DICER1 mutation. She underwent 6 cycles of doxorubicin, ifosfamide, and vincristine and is currently NED on follow-up imaging approximately 10 months after chemotherapy was initiated.

In one case report, a patient with SLCT of intermediate differentiation and RMS of FIGO stage 1C was treated with surgery alone and remained with no evidence of disease (NED) at four years after surgery [8]. These authors report two similar case reports with SLCT of intermediate differentiation and RMS where one patient was found to have NED at ten months after unilateral salpingo-oophorectomy (USO) for stage IA disease while another patient with capsular invasion was initially treated with oophorectomy alone but had reoccurrences at months six and ten, at which time she was treated with unspecified adjuvant chemotherapy [8, 25, 26].

Another case report describes a patient who after a unilateral oophorectomy was found to have an SLCT with focal rhabdomyoblastic differentiation, and she developed an omental mass with features consistent with high-grade sarcoma one year later. She underwent further surgery with a plan for adjuvant chemotherapy but was lost to follow-up [27]. Chougule et al. report a case where ovarian SLCT with RMS and borderline mucinous neoplasm was found in a 26-year-old patient after USO but no further information on treatment or follow-up was provided [28].

Zaloudek and Norris described a 16-year-old patient with stage Ia poorly differentiated SLCT with RMS elements who was treated with USO and later thiotepa and 5-fluorouracil after recurrence at 1.4 years. She was found to have peritoneal metastases on autopsy after her death four months later [29].

The most recent case report of SLCT with RMS elements describes a 70-year-old patient who was diagnosed with stage Ia disease and underwent hysterectomy with left salpingo-oophorectomy (LSO), appendectomy, and omentectomy without evidence of macroscopic disease after the procedures. At 7 months postoperatively, she represented with a large abdominal mass, ascites, unilateral hydronephrosis, and a massive pulmonary embolism. She was treated palliatively and died 15 days later without subsequent autopsy performance [30].

The largest number of case reports was presented by Prat et al. who document nine cases of SLCT with skeletal muscle elements. With one exception, all of these subjects were initially treated with surgery alone. Of those with available information, all subjects had recurrence within one year of initial treatment. Most subjects had recurrence surgically treated and most were deceased within one year. For full information on these subjects, see Table 2 [31].

*Discussion*. Herein we describe 19 cases of SLCT with RMS elements as discussed in prior case reports as well as three cases from the authors' institution. Of these 19 cases, five were lost to follow-up and two were documented as being NED after surgical intervention alone. Of the remaining cases, eleven patients had recurrence within one year and five of these patients were deceased within that year. Of those patients not surgically cured, only three patients were documented as living beyond two years, and all three of these patients received chemotherapy.

In patients with RMS, chemotherapy regimens considered in recurrent disease include vincristine, doxorubicin, and cyclophosphamide; ifosfamide and etoposide; and vincristine, irinotecan, and temozolomide [32, 33]. Standard adjuvant chemotherapy regimens include cisplatin based combinations with etoposide/bleomycin or etoposide/ifosfamide [6]. The three patients presented from our institution with concurrent SLCT and RMS all had clinical evidence of response to chemotherapy that is traditionally used for RMS on follow-up imaging. In conclusion, chemotherapy with doxorubicin and ifosfamide has activity in patients with concurrent SLCT and RMS as does salvage chemotherapy with vincristine, irinotecan, and temozolomide.

## Figures and Tables

**Table 1:** TMN and FIGO classifications for ovarian tumors [23, 24].

TMN	FIGO	
Primary tumor (T)		
T0		No evidence of primary tumor
T1	I	Tumor limited to the ovaries
T1a	IA	Tumor limited to one ovary; capsule intact, no tumor on ovarian surface; no malignant cells in ascites or peritoneal washings
T1b	IB	Tumor limited to both ovaries; capsules intact, no tumor on ovarian surface; no malignant cells in ascites or peritoneal washings
T1c	IC	Tumor limited to one or both ovaries with any of the following: capsule ruptured, tumor on ovarian surface, and malignant cells in ascites or peritoneal washings
T2	II	Tumor involves one or both ovaries with pelvic extension
T2a	IIA	Extension and/or implants on the uterus and/or tube(s); no malignant cells in ascites or peritoneal washings
T2b	IIB	Extension to and/or implants in other pelvic tissues; no malignant cells in ascites or peritoneal washings
T2c	IIC	Pelvic extension and/or implants (T2a or T2b) with malignant cells in ascites or peritoneal washings
T3	III	Tumor involves one or both ovaries with microscopically confirmed peritoneal metastasis outside the pelvis
T3a	IIIA	Microscopic peritoneal metastasis beyond the pelvis (no macroscopic tumor)
T3b	IIIB	Macroscopic peritoneal metastasis beyond the pelvis 2 cm or less in greatest dimension
T3c	IIIC	Macroscopic peritoneal metastasis beyond the pelvis >2 cm in greatest dimension and/or regional lymph node metastasis

Regional lymph nodes (N)		
NX		Regional lymph nodes cannot be assessed
N0		No regional lymph node metastasis
N1	IIIC	Regional lymph node metastasis

Distant metastasis (M)		
M0		No distant metastasis
M1	IV	Distant metastasis (excludes peritoneal metastasis)

**Table 2:** Summary of cases of patients with SLCT and with RMS elements.

Case number/case report author	Age	Stage	Initial treatment	Outcome
1/Plastini	32	Ia	Local resection	Recurrence at 5 months, then radical hysterectomy followed by 2 cycles of doxorubicin/ifosfamide/vincristine, and then 4 cycles of etoposide/ifosfamide. 10 months later recurrence leads to 3 cycles of BEP. Bleomycin was held for cycles 4 through 8. At 3 and 11 months later it had recurrence treated with radiation. Recurrence at 5 months leads to hospice

2/Plastini	30	Ia	Local resection	Lost to follow-up for 13 months and then repeat surgery for recurrence. Two months later, given 6 cycles of total of doxorubicin/ifosfamide/vincristine. Repeat recurrence, given 6 cycles of irinotecan/temozolomide/vincristine. Further progression with small bowel obstruction requiring debulking surgery and ultimately hospice care

3/Plastini	23	Ia	Debulking surgery, USO, and appendectomy	Sp 6 cycles of doxorubicin, ifosfamide, and vincristine and is currently NED (10 months)

4/Grove	29	Ic	USO	NED at 4 years

5/Guerard	16	Ic	Unilateral oophorectomy	Abdominal deposits at 6–10 months

6/Kostopoulou	22	Ia	Unilateral oophorectomy	NED at 10 months

7/Rekhi	17	Ia	TAH-BSO, omentectomy	Omental deposits at 1 year, lost to follow-up

8/Chougule	23	NS	USO	Unknown

9/Zaloudek	16	Ia	USO	Treated with thiotepa and 5-fluorouracil after recurrence at 1.4 years. Died 4 months later with peritoneal metastases on autopsy

10/Papler	70	Ia	Hysterectomy with LSO, appendectomy, omentectomy, and pelvic and para-aortic lymphadenectomy	Recurrence at 7 months with palliative treatment for symptoms

11/Prat	32	Ia	TAH-RSO	Died 5 months later, found to have peritoneal recurrence

12/Prat	22	Unknown	Unknown	Unknown

13/Prat	17	Ia	LSO	Recurrence in R ovary and pelvis at 6 months leads to TAH-RSO and omentectomy. Second recurrence 3 months later treated with melphalan and resection. Died at 1 year

14/Prat	20	IIa	TAH-BSO	At 5 months found to have peritoneal recurrence treated with resection, doxorubicin, and radiation (7600r.) Died at 10 months

15/Prat	36	IIb	RSO	At 5 months found to have peritoneal recurrence and bowel obstruction. Died at 6 months

16/Prat	16	Ia	RSO	Recurrence in left ovary at 6 months treated with TAH-LSO. Second recurrence at 5 years found in peritoneum treated with resection and questionable chemotherapy. Died at 7 years

17/Prat	20	IIb	RSO, intraabdominal BCG, VAC, and doxorubicin	At 4 months recurrence in cul-de-sac and left ovary treated with TAH-LSO and resection. Second recurrence at 7 months in rectovaginal septum treated with radiation (4600r.) Died at 18 months

18/Prat	48	Ia	TAH-LSO	Not documented

19/Prat	23	Ia	RSO	Recurrence in pelvis and peritoneum at years 1 and 2, respectively, treated with unspecified chemotherapy. Patient alive with tumor at 2-year follow-up
